# Methods for Using Race and Ethnicity in Prediction Models for Lung Cancer Screening Eligibility

**DOI:** 10.1001/jamanetworkopen.2023.31155

**Published:** 2023-09-18

**Authors:** Rebecca Landy, Isabel Gomez, Tanner J. Caverly, Kensaku Kawamoto, M. Patricia Rivera, Hilary A. Robbins, Corey D. Young, Anil K. Chaturvedi, Li C. Cheung, Hormuzd A. Katki

**Affiliations:** 1 Division of Cancer Epidemiology and Genetics, National Cancer Institute, National Institutes of Health, Department of Health and Human Services, Bethesda, Maryland; 2 Biostatistics Department, University of Michigan, Ann Arbor; 3 Department of Learning Health Sciences, University of Michigan Medical School, Ann Arbor; 4 Department of Biomedical Informatics, University of Utah, Salt Lake City; 5 Division of Pulmonary and Critical Care Medicine and Wilmot Cancer Institute, University of Rochester, Rochester, New York; 6 Genomic Epidemiology Branch, International Agency for Research on Cancer, Lyon, France; 7 Department of Microbiology, Biochemistry and Immunology, Morehouse School of Medicine, Atlanta, Georgia

## Abstract

**Importance:**

Using race and ethnicity in clinical prediction models can reduce or inadvertently increase racial and ethnic disparities in medical decisions.

**Objective:**

To compare eligibility for lung cancer screening in a contemporary representative US population by refitting the life-years gained from screening–computed tomography (LYFS-CT) model to exclude race and ethnicity vs a counterfactual eligibility approach that recalculates life expectancy for racial and ethnic minority individuals using the same covariates but substitutes White race and uses the higher predicted life expectancy, ensuring that historically underserved groups are not penalized.

**Design, Setting, and Participants:**

The 2 submodels composing LYFS-CT NoRace were refit and externally validated without race and ethnicity: the lung cancer death submodel in participants of a large clinical trial (recruited 1993-2001; followed up until December 31, 2009) who ever smoked (n = 39 180) and the all-cause mortality submodel in the National Health Interview Survey (NHIS) 1997-2001 participants aged 40 to 80 years who ever smoked (n = 74 842, followed up until December 31, 2006). Screening eligibility was examined in NHIS 2015-2018 participants aged 50 to 80 years who ever smoked. Data were analyzed from June 2021 to September 2022.

**Exposure:**

Including and removing race and ethnicity (African American, Asian American, Hispanic American, White) in each LYFS-CT submodel.

**Main Outcomes and Measures:**

By race and ethnicity: calibration of the LYFS-CT NoRace model and the counterfactual approach (ratio of expected to observed [E/O] outcomes), US individuals eligible for screening, predicted days of life gained from screening by LYFS-CT.

**Results:**

The NHIS 2015-2018 included 25 601 individuals aged 50 to 80 years who ever smoked (2769 African American, 649 Asian American, 1855 Hispanic American, and 20 328 White individuals). Removing race and ethnicity from the submodels underestimated lung cancer death risk (expected/observed [E/O], 0.72; 95% CI, 0.52-1.00) and all-cause mortality (E/O, 0.90; 95% CI, 0.86-0.94) in African American individuals. It also overestimated mortality in Hispanic American (E/O, 1.08, 95% CI, 1.00-1.16) and Asian American individuals (E/O, 1.14, 95% CI, 1.01-1.30). Consequently, the LYFS-CT NoRace model increased Hispanic American and Asian American eligibility by 108% and 73%, respectively, while reducing African American eligibility by 39%. Using LYFS-CT with the counterfactual all-cause mortality model better maintained calibration across groups and increased African American eligibility by 13% without reducing eligibility for Hispanic American and Asian American individuals.

**Conclusions and Relevance:**

In this study, removing race and ethnicity miscalibrated LYFS-CT submodels and substantially reduced African American eligibility for lung cancer screening. Under counterfactual eligibility, no one became ineligible, and African American eligibility increased, demonstrating the potential for maintaining model accuracy while reducing disparities.

## Key Points

**Question:**

What approaches to using race and ethnicity in the life-years gained from screening–computed tomography (LYFS-CT) prediction model, which is used to inform lung cancer screening eligibility, can best identify high-benefit individuals and minimize racial and ethnic disparities in screening eligibility?

**Findings:**

This diagnostic study found that removing race and ethnicity curtailed the ability of LYFS-CT to identify high-benefit racial and ethnic minority individuals and reduced screening eligibility for African American individuals while eligibility for other groups increased. The counterfactual eligibility model was found to increase eligibility, maintain predictive accuracy across all racial and ethnic groups, and avoid excluding individuals from screening based on racial and ethnic minority status.

**Meaning:**

These findings suggest that thoughtful use of race and ethnicity data in prediction models may help reduce disparities.

## Teaser

This diagnostic study uses data for individuals who ever smoked to examine 2 approaches for incorporating race and ethnicity in a lung cancer prediction model and determine changes in screening eligibility.

## Introduction

The 2021 United States Preventive Services Task Force (USPSTF) lung cancer screening recommendations expanded eligibility to “help partially ameliorate racial disparities in screening eligibility.”^1,2^ However, the recommendations remain anchored on binary age/pack-year/quit-year cut points that do not account for racial and ethnic differences in lung cancer risk, potentially perpetuating health disparities.^3,4,5^ In particular, African American individuals have higher risks of lung cancer, despite smoking less than White individuals, and develop cancer at younger ages.^6^ Racial and ethnic differences can be accounted for in prediction models, with numerous studies suggesting that prediction models identify large groups of high-benefit racial minority individuals (especially African American individuals) who are currently ineligible for screening under USPSTF recommendations.^7,8^ Augmenting USPSTF criteria with prediction models has been recommended by the National Comprehensive Cancer Network,^9^ American College of Chest Physicians (ACCP),^10^ and the American Thoracic Society^5^ as a way to reduce disparities in eligibility.

However, clinical use of race and ethnicity data requires careful consideration about racial and ethnic disparities, with numerous examples of clinical rules based on flawed data or biased presumptions that reduce access to health care and exacerbate disparities.^11^ For example, race-specific rules for defining kidney disease using estimated glomerular filtration rate and obstructive pulmonary disease using forced expiratory volume in 1 second have had the direct consequence of avoiding disease diagnosis and therefore medical care on the basis of race.^12,13^ Medical societies have therefore advocated to remove racial and ethnic information from clinical rules because of these concerns.^14,15,16,17^

Decisions based on clinical prediction models may reduce disparities when race and ethnicity are independent predictors of outcome risk, as for cardiovascular disease, lung cancer, and diabetes.^8,18,19^ This is a result of providing more accurate estimates for individuals of each race and ethnicity when race and ethnicity are included in the model because models developed mainly using data from White individuals reflect only their experience. For screening, which involves asymptomatic individuals, the harms must not exceed the benefits. Individuals with little chance of benefiting nonetheless remain susceptible to the harms of screening.^20^ Thus, simply making more racial minority individuals eligible, for example, by lowering the eligibility risk- or benefit-threshold for individuals from certain races and ethnicities, could be a poor strategy for addressing disparities if individuals have low benefit. Furthermore, screening guidelines typically recommend 5 to 10 years’ minimum life expectancy^21,22^ to ensure that individuals are healthy enough for cancer treatments and are not likely to die soon from comorbid conditions. However, some groups may have lower life expectancy due to lack of access to care or historic discrimination, and it may be unfair to penalize individuals who are projected to have low life expectancy solely because of their self-reported race and ethnicity.

These considerations highlight the complexity of developing general strategies for using prediction models to improve the fairness of clinical rules. The life-years gained from screening–computed tomography (LYFS-CT) model^23^ individualizes lung cancer mortality risk and life expectancy and is recommended by the ACCP^10^ for supporting personalized shared decision-making for lung cancer screening. LYFS-CT is implemented in the Decision Precision+ clinical decision support tool for lung cancer screening.^24^ LYFS-CT is particularly interesting to examine from the perspective of fairness because, unlike risk models, it explicitly incorporates life expectancy. Here, we examined the effect of different approaches to incorporating race and ethnicity in the LYFS-CT model on screening eligibility for a contemporary representative US population.

## Methods

The National Institutes of Health Office of Human Subjects Research deemed this study exempt from institutional review board approval. This report follows the Transparent Reporting of a Multivariable Prediction Model for Individual Prognosis or Diagnosis (TRIPOD) reporting guideline. Data were analyzed from June 2021 to September 2022.

### Model Development and Validation

#### Life-Years Gained From Attending Screening

The LYFS-CT model^23^ models the days of life gained from attending screening akin to the National Lung Screening Trial (3 annual low-dose CT screens). (See the eMethods in Supplement 1 for more details on the LYFS-CT.) This model comprises (1) an all-cause mortality (ACM) submodel and (2) a lung cancer death risk submodel called the lung cancer death risk assessment tool (LCDRAT). The life gained from attending screening is calculated as the difference in life expectancy when attending screening and not attending screening, where attending screening reduces the risk of lung cancer death by 20.4% each year for 5 years, according to National Lung Screening Trial findings.^25^ LYFS-CT differs from risk models because it can identify younger healthier people who may live decades if their life is saved by screening.^23^ Focusing on LYFS-CT rather than a risk model enables us to examine the effect of race and ethnicity on both risk and life expectancy, both of which are important in clinical practice.

#### Lung Cancer Death Risk

We refit our 5-year LCDRAT model^26^ using a Cox proportional hazards model with age as the time scale, removing race from both its lung cancer death and the competing risk components (LCDRAT-NoRace). The model includes self-reported smoking variables as well as demographic variables. This model was developed using data from 39 180 individuals aged 55 to 74 years in the control group of the Prostate, Lung, Colorectal, and Ovarian Cancer Screening Trial (PLCO)^27^ who ever smoked, of whom 1092 died of lung cancer. Individuals were recruited into PLCO in the United States from 1993 to 2001, with follow-up data available until December 31, 2009. Individuals were censored at the earliest of death from lung cancer, death from other causes, or end of follow-up. Details on all the data sets used in this study are given in eTable 1 in Supplement 1. Not everyone eligible for the PLCO would qualify for screening under USPSTF 2021 recommendations (for example, if they had smoked <20 pack-years); LCDRAT should therefore be valid for individuals who would not be eligible under current recommendations.

We validated the LCDRAT-NoRace model using National Health Interview Survey (NHIS) data from 1997 to 2001, linked to the National Death Index, providing cause-of-death data up to December 31, 2006. The NHIS is an annual cross-sectional, nationally representative survey of the noninstitutionalized US population. We included 28 232 individuals aged 50 to 80 years with known cause of death and without prior lung cancer who ever smoked (673 lung cancer deaths). Individuals were censored at the earliest of death from lung cancer, death from other causes, or end of follow-up. Multiple imputation was carried out to account for missing data (eMethods in Supplement 1). Model validity was assessed using calibration (ratio of observed to expected lung cancer deaths within 5 years) and predictiveness (area under the curve) for all individuals and stratified by race and ethnicity.

To create the LYFS-CT NoRace model, we used the refit LCDRAT-NoRace model and also refit the ACM submodel removing race and ethnicity, which was developed (odd years; n = 74 842; 16 363 deaths) and validated (even years; n = 72 199; 14 003 deaths) using data from NHIS 1997-2014 individuals aged 40 to 80 years who had ever smoked, linked to mortality data up to December 2015. Individuals were censored at the earliest of end of follow-up or death. Multiple imputation was carried out to account for missing data (eMethods in Supplement 1).

### Counterfactual Eligibility

Classifying a person as ineligible for screening because of lower estimated life expectancy may perpetuate historical disparities if their lower life expectancy results from unequal access to health care or social/economic marginalization more broadly. Therefore, we also considered counterfactual eligibility, where race and ethnicity remain in the model and life expectancy is calculated in the absence of screening both for non-White individuals as well as individuals with the same covariate values but of White race; the higher value for life gained from screening is used to inform screening eligibility (LYFS-CT CounterfactualACM). The LYFS-CT CounterfactualACM model allows an ineligible person to become eligible if the only reason for ineligibility was low life expectancy due to their race or ethnicity. We additionally recalculated both lung cancer death risk and life expectancy in the absence of screening for White individuals with the same covariate values (LYFS-CT Counterfactual). Importantly, while removing race and ethnicity can change an individual’s eligibility status in either direction, applying counterfactual eligibility can only make ineligible individuals eligible.

### Statistical Methods

We compared the number eligible for screening from each race and ethnicity under the following models: (1) the standard LYFS-CT model, (2) removing race from only the ACM model component of LYFS-CT (LYFS-CT NoRaceACM), (3) removing race from both the lung cancer death risk (LCDRAT) component of LYFS-CT and the ACM model component of LYFS-CT (LYFS-CT NoRace), (4) taking the higher life-gained benefit from LYFS-CT NoRaceACM for racial and ethnic minority individuals using their own race and ethnicity or for a similar individual who is White (LYFS-CT CounterfactualACM), and (5) taking the higher life-gained benefit from LYFS-CT NoRace using their own race and ethnicity or for a similar White individual (LYFS-CT Counterfactual). When we refer to Asian American individuals, this group does not include American Indian, Alaska Native, Native Hawaiian, or Other Pacific Islander individuals.

We defined an individual as high benefit and therefore eligible for screening if they met the ACCP eligibility criteria of at least 16.2 days of life gained from attending screening.^10^ We applied these models to the NHIS 2015-2018 data and report the number eligible and the proportion of ever-smokers aged 50 to 80 years from that race and ethnicity who would be eligible. Race and ethnicity were self-reported; individuals were asked if they were of Hispanic ethnicity (yes, no) and which race they were (American Indian or Alaskan Native, Asian only, Black/African American only, White only, or multiple race). We also calculated the number and proportion of preventable lung cancer deaths and gainable years of life, assuming the original LCDRAT and LYFS-CT models are correct for individuals of all races and ethnicities.

Analyses were carried out in R version 4.2 using the survey package to account for the NHIS sample design. Multiple imputation was carried out to account for missing data in NHIS 2015-2018 (eMethods in Supplement 1).

## Results

The NHIS 2015-2018 contained 25 601 individuals aged 50 to 80 years who had ever smoked, including 2769 African American individuals, 649 Asian American individuals, 1855 Hispanic American individuals, and 20 328 White individuals. Of the races and ethnicities considered, among individuals in the United States who ever smoked, African American individuals are most likely to be currently smoking, but White individuals have smoked the most pack-years (Table 1). According to LYFS-CT, after accounting for other lung cancer risk factors and 12 comorbid conditions, African American individuals have elevated risks of lung cancer death (hazard ratio [HR], 1.48; 95% CI, 1.17-1.87) and ACM (HR, 1.22; 95% CI, 1.15-1.31) compared with White individuals; Hispanic American individuals (lung cancer death HR, 0.69, 95% CI, 0.40-1.19; ACM HR, 0.87; 95% CI, 0.80-0.94) and Asian American individuals (lung cancer death HR, 0.66; 95% CI, 0.45-0.97; ACM HR, 0.75; 95% CI, 0.64-0.87) have lower risks for both (Table 2).

**Table 1.** Characteristics of Individuals Aged 50 to 80 Years Who Ever Smoked Using the Sample Weights in the 2015-2018 National Health Interview Survey

Characteristic	No. (%)				
	African American	Asian American	Hispanic American	Non-Hispanic White	Total
Overall (row %)	4 301 164 (10)	1 275 950 (3)	3 866 398 (9)	34 973 814 (79)	44 417 326 (100)
Sex					
Male	2 271 620 (53)	961 482 (75)	2 361 660 (61)	18 395 133 (53)	23 989 895 (54)
Female	2 029 544 (47)	314 467 (25)	1 504 738 (39)	16 578 681 (47)	20 427 430 (46)
Age, mean (IQR), y	62 (56-68)	62 (55-68)	61 (54-67)	63 (56-70)	63 (56-69)
50-54	823 232 (19)	277 678 (22)	1 017 249 (26)	6 134 400 (18)	8 252 559 (19)
55-59	939 813 (22)	264 578 (21)	874 089 (23)	6 860 064 (20)	8 938 545 (20)
60-64	957 042 (22)	253 506 (20)	733 859 (19)	6 868 729 (20)	8 813 136 (20)
65-69	707 622 (16)	202 080 (16)	547 941 (14)	6 170 666 (18)	7 628 308 (17)
70-74	455 178 (11)	168 989 (13)	425 135 (11)	5 082 108 (15)	6 131 410 (14)
75-80	418 278 (10)	109 119 (9)	268 126 (7)	3 857 846 (11)	4 653 368 (10)
Smoking status					
Current smoker	1 832 917 (43)	417 573 (33)	1 230 466 (32)	10 679 915 (31)	14 160 870 (32)
Former smoker	2 468 247 (57)	858 377 (67)	2 635 932 (68)	24 293 899 (69)	30 256 455 (68)
Self-reported emphysema status					
Emphysema	157 131 (4)	36 058 (3)	109 837 (3)	2 013 301 (6)	2 316 327 (5)
No emphysema	4 144 034 (96)	1 239 891 (97)	3 756 561 (97)	32 960 513 (94)	42 100 999 (95)
Quit years (% of former smokers)					
<1 y	110 135 (4)	20 005 (2)	64 125 (2)	736 818 (3)	931 083 (3)
1-4 y	268 674 (11)	90 315 (11)	213 835 (8)	2 141 715 (9)	2 714 538 (9)
5-9 y	274 832 (11)	50 791 (6)	254 435 (10)	2 122 091 (9)	2 702 149 (9)
≥10 y	1 814 606 (74)	697 265 (81)	2 103 538 (80)	19 293 275 (79)	23 908 684 (79)
Current smokers’ pack-years (% of current smokers)					
<20	1 150 995 (63)	271 679 (65)	831 905 (68)	3 677 570 (34)	5 932 149 (42)
20-29	340 365 (19)	76 999 (18)	193 514 (16)	2 082 079 (19)	2 692 957 (19)
30-49	221 682 (12)	52 658 (13)	139 108 (11)	3 330 511 (31)	3 743 960 (26)
50-69	83 144 (5)	12 185 (3)	38 715 (3)	1 023 557 (10)	1 157 601 (8)
≥70	36 731 (2)	4051 (1)	27 225 (2)	566 197 (5)	634 203 (4)
Former smokers’ pack-years (% of former smokers)					
<20	1 593 891 (65)	579 438 (68)	1 838 195 (70)	15 400 255 (63)	19 411 778 (64)
20-29	309 102 (13)	88 960 (10)	281 156 (11)	3 092 972 (13)	3 772 190 (12)
30-49	366 975 (15)	122 932 (14)	336 064 (13)	3 606 497 (15)	4 432 467 (15)
50-69	110 822 (4)	31.243 (4)	105 537 (4)	1 152 825 (5)	1 400 427 (5)
≥70	87 459 (4)	35 805 (4)	74 980 (3)	1 041 350 (4)	1 239 593 (4)
Body mass index^a^					
<18.5	54 571 (1)	22 441 (2)	27 780 (1)	525 803 (2)	630 595 (1)
18.5-25	1 075 554 (25)	579 360 (45)	804 772 (21)	9 826 538 (28)	12 286 225 (28)
25-30	1 420 171 (33)	466 364 (37)	1 592 324 (41)	12 891 014 (37)	16 369 873 (37)
≥30	1 750 868 (41)	207 784 (16)	1 441 522 (37)	11 730 458 (34)	15 130 633 (34)

^a^ Calculated as weight in kilograms divided by height in meters squared.

**Table 2.** Submodels for Lung Cancer Death and All-Cause Mortality Within LYFS-CT

Factor	Coding	HR (95% CI)^a^			
		All-cause mortality	All-cause mortality without race or ethnicity	Lung cancer death	Lung cancer death without race or ethnicity
3 Annual CT screens	Binary	NI	NI	0.80 (0.69-0.92)^b^	0.80 (0.69-0.92)^b^
Age	Log term	NI^c^	NI^c^	422.4 (181.10-985.19)	418.15 (179.30-975.17)
Sex (female)	Binary	0.72 (0.69-0.75)	0.73 (0.70-0.76)	0.84 (0.74-0.95)	0.84 (0.74-0.95)
Race and ethnicity (self-reported)	Categorical				
	African American, non-Hispanic	1.22 (1.15-1.31)		1.48 (1.17-1.87)	
	Asian or other	0.75 (0.64-0.87)		0.66 (0.45-0.97)	
	Hispanic	0.87 (0.80-0.94)		0.69 (0.40-1.19)	
	White, non-Hispanic	1 [Reference]	NI	1 [Reference]	NI
Education^d^	Trend	0.93 (0.92-0.94)	0.93 (0.91-0.94)	0.91 (0.87-0.94)	0.91 (0.87-0.94)
Calendar year assessed	Linear	0.98 (0.98-0.99)	0.98 (0.98-0.99)	NI	NI
BMI ≤18.5^e^	Binary	NI	NI	1.42 (0.90-2.26)	1.45 (0.91-2.30)
BMI	Log term	NI	NI	0.45 (0.30-0.68)	0.48 (0.32-0.72)
	Categorical			NI	NI
	20-<25	1 [Reference]	1 [Reference]		
	≤18.5	1.92 (1.71-2.17)	1.91 (1.69-2.15)		
	18.5-<20	1.34 (1.21-1.47)	1.32 (1.20-1.46)		
	25-<30	0.82 (0.78-0.86)	0.82 (0.79-0.86)		
	30-<35	0.87 (0.82-0.92)	0.87 (0.82-0.92)		
	>35	1.02 (0.94-1.09)	1.03 (0.95-1.11)		
Pack-years	Categorical	NI	NI		
	0-<30			1 [Reference]	1 [Reference]
	30-<40			1.74 (1.35-2.25)	1.72 (1.34-2.22)
	40-<50			2.11 (1.68-2.66)	2.09 (1.66-2.62)
	≥50			2.45 (1.86-3.22)	2.42 (1.84-3.18)
	Square root term	1.04 (1.02-1.06)	1.04 (1.02-1.06)		
Quit-years	Log term^f^	0.82 (0.80-0.84)	0.82 (0.80-0.84)	0.69 (0.64-0.74)	0.68 (0.64-0.73)
Years smoked	Log term	NI	NI	1.39 (1.10-1.77)	1.41 (1.11-1.78)
>1 Pack/d	Binary	NI	NI	1.27 (1.05-1.54)	1.25 (1.03-1.51)
Cigarettes/d	Log term	1.09 (1.03-1.16)	1.08 (1.02-1.15)	NI	NI
Lung cancer family history^g^	Trend	NI	NI	1.42 (1.25-1.63)	1.43 (1.25-1.63)
Uses special equipment^h^	Binary	1.66 (1.57-1.76)	1.68 (1.59-1.78)	NI	NI
Liver condition past 1 y	Binary	1.68 (1.49-1.90)	1.69 (1.49-1.91)	NI	NI
Emphysema	Binary	1.57 (1.46-1.68)	1.56 (1.45-1.67)	1.74 (1.45-2.08)	1.74 (1.45-2.08)
Diabetes	Binary	1.47 (1.39-1.55)	1.46 (1.39-1.54)	NI	NI
Weak/failing kidneys past 1 y	Binary	1.41 (1.27-1.57)	1.41 (1.27-1.57)	NI	NI
Prior cancer	Binary	1.24 (1.18-1.31)	1.24 (1.18-1.30)	NI	NI
Prior stroke	Binary	1.29 (1.21-1.39)	1.30 (1.21-1.39)	NI	NI
Prior heart attack	Binary	1.25 (1.17-1.34)	1.25 (1.17-1.34)	NI	NI
Coronary heart disease	Binary	1.13 (1.05-1.22)	1.13 (1.05-1.22)	NI	NI
Heart disease	Binary	1.15 (1.09-1.21)	1.15 (1.09-1.21)	NI	NI
Chronic bronchitis past 1 y	Binary	1.11 (1.04-1.19)	1.11 (1.04-1.19)	NI	NI
Hypertension	Binary	1.16 (1.11-1.21)	1.17 (1.13-1.22)	NI	NI
Angina pectoris	Binary	1.01 (0.94-1.08)	1.00 (0.93-1.07)	NI	NI

Abbreviations: BMI, body mass index; CT, computed tomography; HR, hazard ratio; LYFS-CT, life-years gained from screening–computed tomography; NI, not included as a covariate.

^a^ Hazard ratios for all-cause mortality were updated using additional National Health Interview Survey data to allow more racial and ethnic minority individuals to be included, though are similar to those previously published.

^b^ Represents the rate ratio observed in the National Lung Screening Trial over 5 years of follow-up.

^c^ Age is used as the time scale for the overall mortality model.

^d^ <12 Grade = 1, high school graduate = 2, post high school but no college = 3, some college = 4, bachelor’s degree = 5, graduate school = 6.

^e^ Calculated as weight in kilograms divided by height in meters squared.

^f^ Natural logarithm of ≥1 quit-years.

^g^ First-degree relatives (siblings, parents, children) with history of lung cancer; none with lung cancer = 0, 1 with lung cancer = 1, ≥2 with lung cancer = 2.

^h^ Individuals with health problems that require use of special equipment, such as a cane, wheelchair, special bed, or special telephone.

After each LYFS-CT submodel was refit without race and ethnicity, the other parameters changed minimally (Table 2). However, because the cohorts used to develop these risk models are predominantly non-Hispanic White individuals (PLCO: 86%; NHIS 1997-2014: 64%), removing race and ethnicity from risk models means that the new models more closely mirror the White experience. Without race and ethnicity, the new lung cancer death risk submodel (LCDRAT-NoRace) underestimated risk in African American individuals by 28% (ratio of expected to observed number of outcomes [E/O] of 0.72; 95% CI, 0.52-1.00), and possibly overestimated risk in Hispanic American individuals and Asian American individuals by 22% to 25%, and calibration for White individuals was almost unchanged (Table 3). Removing race and ethnicity from the ACM submodel underestimated mortality in African American individuals by 10% (E/O, 0.90; 95% CI, 0.86-0.94), and overestimated mortality in Hispanic American individuals (E/O, 1.08, 95% CI, 1.00-1.16) and Asian American individuals (E/O, 1.14, 95% CI, 1.01-1.30), and calibration for White individuals was again virtually unchanged (Table 3). Removing race and ethnicity hardly reduced the area under the curve for the submodels (eTable 2 in Supplement 1). The pattern of calibration of ACM submodels by risk quintile and by race and ethnicity reflects the overall estimate except in the lowest quintile (eTable 3 in Supplement 1), where Hispanic American mortality is underestimated (including race and ethnicity: E/O, 0.50; 95% CI, 0.32-0.78; excluding race and ethnicity: E/O, 0.57; 95% CI, 0.34-0.94). There were insufficient cases to assess calibration of the lung cancer mortality models by risk quintile and race and ethnicity.

**Table 3.** Calibration of LYFS-CT Submodels for Risk of Lung Cancer Death and Risk of All-Cause Mortality

	No. of lung cancer deaths^a^	E/O (95% CI)		No. of all-cause deaths	E/O (95% CI)	
		Current lung cancer death model	Lung cancer death model without race and ethnicity		Current all-cause mortality model	All-cause mortality model without race and ethnicity
Total	673	0.93 (0.82-1.04)	0.92 (0.82-1.04)	30 462	0.98 (0.97-1.00)	0.99 (0.97-1.00)
Race and ethnicity						
African American	98	1.03 (0.74-1.44)	0.72 (0.52-1.00)	3424	1.03 (0.98-1.08)	0.90 (0.86-0.94)
Asian American	4	0.80 (0.26-2.47)	1.22 (0.40-3.74)	527	0.91 (0.80-1.04)	1.14 (1.01-1.30)
Hispanic American	31	0.85 (0.50-1.46)	1.25 (0.73-2.15)	2658	0.96 (0.90-1.03)	1.08 (1.00-1.16)
White	534	0.92 (0.81-1.05)	0.93 (0.82-1.06)	18 111	0.98 (0.96-1.00)	0.99 (0.97-1.00)

Abbreviations: E/O, ratio of expected number of outcomes to observed number of outcomes; LYFS-CT, life-years gained from screening–computed tomography.

^a^ Analyses by race and ethnicity excluded 6 deaths among individuals who did not self-report as African American, Asian American, Hispanic American, or White.

**Figure 1.**
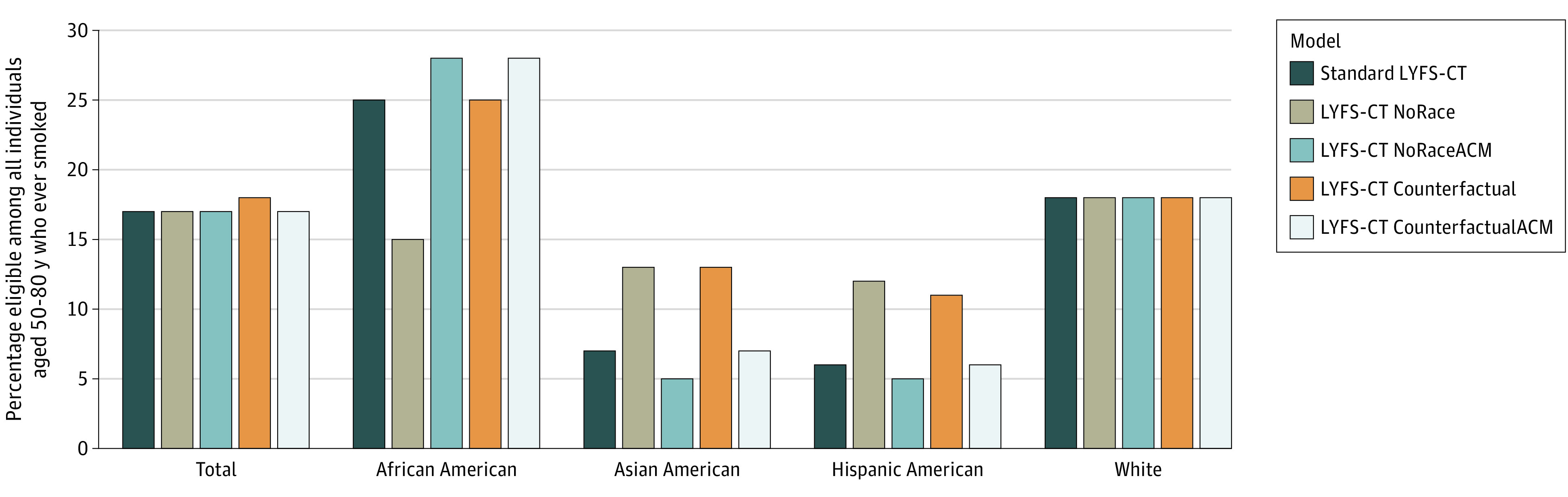
Individuals Aged 50 to 80 Years Who Ever Smoked Who Are Eligible for Lung Cancer Screening Percentage of individuals aged 50 to 80 years who ever smoked, stratified by race and ethnicity, who would be eligible for lung cancer screening at a threshold of 16.2 days of life gained under the life-years gained from screening–computed tomography (LYFS-CT) model for the 5 models considered. ACM indicates all-cause mortality; Counterfactual describes the submodels that set race and ethnicity to White; NoRace, the submodels that removed race and ethnicity.

**Figure 2.**
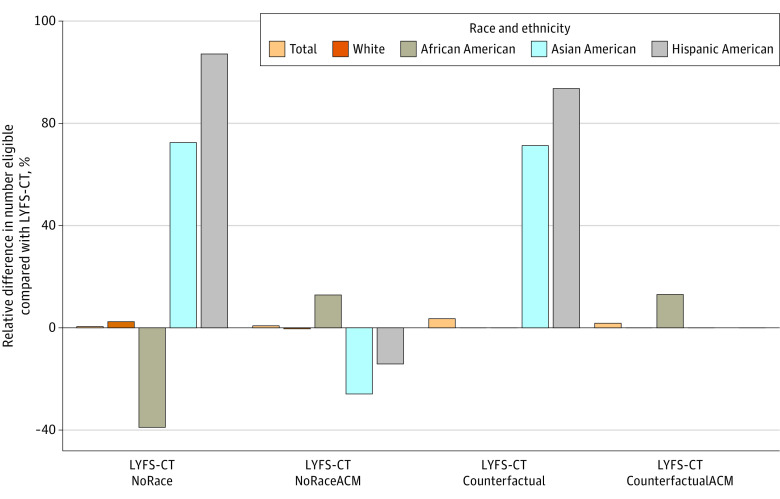
Difference in the Number Eligible for Screening Relative to the Standard Life-Years Gained From Screening–Computed Tomography (LYFS-CT) Model Percentage difference in the number eligible for screening within each racial and ethnic group for each of the 4 models considered relative to the standard LYFS-CT model. ACM indicates all-cause mortality; Counterfactual describes the submodels that set race and ethnicity to White; NoRace, the submodels that removed race and ethnicity.

In the standard LYFS-CT model, 17% of US individuals aged 50 to 80 years who ever smoked (n = 7 574 448) would be eligible for screening (Figure 1 and eTable 4 in Supplement 1). The proportion eligible by race and ethnicity ranged from 6% of Hispanic American individuals to 25% of African American individuals. Removing race and ethnicity entirely from LYFS-CT (LYFS-CT NoRace) would substantially alter eligibility for racial and ethnic minority groups: reducing African American eligibility by 413 813 individuals (39.1% reduction), replacing them with 233 604 Hispanic American individuals and 67 891 Asian American individuals who ever smoked (107.6% and 72.8% increases, respectively). However, according to the standard LYFS-CT model, the African American individuals who became ineligible would gain more life-years from screening than the Hispanic American individuals and Asian American individuals made newly eligible when excluding race and ethnicity in the LYFS-CT NoRace model (18.5 vs 13.5 and 13.9 days of life gained, respectively) (Figure 2 and eTable 4 in Supplement 1).

Under the LYFS-CT NoRaceACM model, an additional 58 075 individuals would be eligible: 136 267 more African American individuals (12.9%) but fewer members of other races (Asian American: 24 285 [26.0% reduction]; Hispanic American: 30 797 [14.2% reduction]; White: 23 109 [0.4% reduction]) (Figure 2 and eTable 4 in Supplement 1). Furthermore, the African American individuals who became eligible would have slightly lower benefit (according to the standard LYFS-CT model) than the Asian American and Hispanic American individuals who would become ineligible (15.5 vs 17.4 and 16.7 days of life gained, respectively) (Figure 2 and eTable 4 in Supplement 1).

Under counterfactual eligibility, by definition, eligibility for White individuals is unchanged and no one can change from being eligible to ineligible (Figure 2 and eTable 4 in Supplement 1). For counterfactual eligibility for only the ACM submodel (LYFS-CT CounterfactualACM), African American eligibility increased by 13% (n = 138 473), while Hispanic American and Asian American eligibility was unchanged. The additional African American individuals had a mean 15.5 days of life gained under the standard LYFS-CT model, close to the 16.2-day threshold. Thus, counterfactual eligibility for only ACM would increase African American eligibility without decreasing eligibility for other racial and ethnic groups.

Under counterfactual eligibility for both lung cancer death and all-cause mortality (LYFS-CT Counterfactual), no additional African American individuals were chosen, because their predicted lung cancer death risk is underestimated when race and ethnicity are removed from the lung cancer death model. Instead, an additional 270 941 individuals would be eligible for screening, of whom 75% would be Hispanic American individuals (an increase of 94.0%), and the remaining 25% would be Asian American individuals (an increase of 71.6%). However, these newly eligible people may gain a mean of only 13.8 days (Hispanic American) and 13.9 days (Asian American) of life from attending screening (according to the standard LYFS-CT model), which is less than the 15.5 days of life gained among African American individuals chosen by the LYFS-CT CounterfactualACM model.

## Discussion

This study found that removing race and ethnicity from the LYFS-CT submodels curtailed the ability to identify racial and ethnic differences in risk in favor of an average risk that more closely reflects the non-Hispanic White individuals who make up the majority of the cohorts in which these models were fitted. Excluding race and ethnicity from the lung cancer death risk model resulted in substantial underestimation of risk for African American individuals and may exclude many high-benefit African American individuals from screening.

Removing race and ethnicity from the ACM model resulted in underestimation of life expectancy for Hispanic American individuals and Asian American individuals; hence, high-benefit Hispanic American individuals and Asian American individuals would become ineligible for screening. Removing race and ethnicity would substantially weaken the ability of LYFS-CT to identify high-benefit racial or ethnic minority individuals and would provide inaccurate risk and benefit estimates, which impairs shared decision-making.

In contrast, counterfactual eligibility preserves model accuracy and ensures that every group can only gain in eligibility. Counterfactual eligibility for only ACM (LYFS-CT CounterfactualACM) increased eligibility for African American individuals who barely missed eligibility (mean 15.5 days of life, close to the 16.2-day threshold) due to lower life expectancy because they are African American. The latest version of LYFS-CT, accessible online^28^ and also in a form that is integrated with electronic health records,^24^ now implements counterfactual eligibility (LYFS-CT CounterfactualACM).

Note that counterfactual eligibility for both lung cancer death and ACM together (LYFS-CT Counterfactual) was unable to identify any additional African American individuals for eligibility. This happened because substituting White race into the model reduced lung cancer death risk, and hence the benefit from screening for African American individuals, which in turn reduced the estimate of life-years gained. This highlights the importance of careful consideration of the quantity to consider as counterfactual.

Counterfactual eligibility, along with the LYFS-CT NoRaceACM model, attempts to account for race and ethnicity reflecting both biological and social constructs. Sometimes risk differs because of biological and genetic differences (eg, sickle cell disease). African American individuals have an increased risk of lung cancer that is, in part, likely due to biological effects of smoking, and increased risk should lead to increased screening eligibility. Cotinine clearance has been shown to be slower in African American individuals compared with White individuals.^29^ Additionally, comparative transcriptomic profiling has shown differences in non–small cell lung cancer tumor biology, indicating at least part of the difference may be biological.^30^

However, differences in risk can also reflect social differences between races and ethnicities and arise due to unmeasured social determinants of health, such as differences in environmental exposures, which it is currently not practical to include in prediction models. Additionally, mechanistic hypotheses include that African American individuals smoke more mentholated cigarettes, which can be inhaled deeper^31,32^; leading them to inhale more nicotine per cigarette smoked (30% more in one study^29^), exposing them to more carcinogens despite smoking a lower number of cigarettes per day, with higher total nicotine equivalents in urine per cigarette compared with White individuals.^33^ Thus, race and ethnicity may often be a marker of risk rather than a true biological risk factor.^34^ Continuous efforts are required to incorporate social determinants of health into prediction models when such information can be more routinely collected in electronic health records.

Given this complexity, decision-makers should not only consider the importance of race and ethnicity for accurately measuring an individual’s risk but also consider how risk estimates will be used to inform decisions. It is especially important to avoid unintentionally perpetuating historical disparities. In the case of lung cancer screening, the lower life expectancy of African American individuals compared with White individuals partly results from lifelong reduced access to care, including a higher proportion being unable to see a doctor because of cost^35^ and both historical and ongoing social and economic marginalization.^36,37,38,39^

We note the importance of carefully considering the circumstances in which a counterfactual approach would be appropriate: when doing so can help reduce racial disparities while not depriving anyone (including White individuals) of their eligibility for services. We found that using the LYFS-CT CounterfactualACM model, where the predicted life expectancy of an individual with the same covariates but White race is also considered, (1) ensures model accuracy, (2) avoids making anyone ineligible because of the impact of their race and ethnicity on life expectancy, and (3) avoids making anyone eligible who has very low lung cancer death risk and therefore has low expected net benefit. Additionally, counterfactual approaches can immediately be applied to existing models, whereas developing new models excluding race and ethnicity would require revamping existing models. However, if using counterfactual eligibility, it is important to ensure that the life-years gained for individuals eligible only under the counterfactual scenario are not much below the accepted threshold because their actual benefit is lower than other eligible people (according to the LYFS-CT model). Alternative ways of incorporating race and ethnicity into estimates of outcome risk and ACM should be empirically examined as approaches to reducing disparities in eligibility.

Because life expectancy is an important consideration for any preventive intervention, our findings on ACM could be relevant to other preventive services. As observed in this study, we expect eligibility for White individuals will be unaffected when removing race from predictions where the data are largely derived from White populations but will result in worse model calibration in racial minority individuals. Because African American individuals and Hispanic American individuals/Asian American individuals have HRs for ACM in opposite directions relative to White individuals, removing race and ethnicity from the ACM model will increase African American eligibility but decrease Hispanic American/Asian American eligibility. In situations where each racial and ethnic minority group has risk-increasing HRs for the outcome of interest, removing race and ethnicity would reduce eligibility for all racial and ethnic minority individuals.

A lung cancer incidence risk model, the PLCO-M2012,^40^ has produced a version excluding race^41^ for use outside the United States. Their conclusions were similar to ours: removing race and ethnicity reduced the proportion of African American individuals who developed lung cancer who would be eligible for screening and increased the proportion for Hispanic American individuals, with minimal impact overall or for White individuals.^41^

We used data that are representative of the contemporary US population to estimate the proportion of individuals who would be eligible for screening in each race and ethnicity. Similarly, we used population-representative data to develop and externally validate the life expectancy submodel.

### Limitations

The main limitation of our study is that our model estimates of life-years gained assume the model containing race and ethnicity is accurate for all individuals. For White and African American individuals, model calibration is good for both the submodels, including across all risk quintiles for ACM; for Asian American and Hispanic American individuals, the confidence intervals around our calibration estimates are wide because of a lack of data, and ACM is underestimated among Hispanic American individuals in the lowest risk quintile.

## Conclusions

In this study, removing race and ethnicity miscalibrated LYFS-CT submodels and substantially reduced African American eligibility for lung cancer screening. Under counterfactual eligibility, no one became ineligible, and African American eligibility increased, demonstrating the potential for maintaining model accuracy while reducing disparities. These findings suggest that when race and ethnicity is truly an independent predictor of risk and its inclusion in prediction models increases predictive accuracy, excluding race and ethnicity can exacerbate racial and ethnic disparities. Race and ethnicity can represent risk pathways, such as environmental factors or other social determinants of health, that are difficult to ascertain in the clinic. Thoughtful use of race and ethnicity data may account for important historical factors that have led to disparities. Thus, appropriate use of prediction models that include race and ethnicity can provide a powerful tool for reducing disparities.

## Notes

Supplement 1.
eMethods.
**eTable 1.** Data sources used to develop and validate the lung cancer death risk and all-cause mortality models **eTable 2.** AUC of LYFS-CT submodels for risk of lung-cancer death and risk of all-cause mortality **eTable 3.** Calibration (ratio of expected number of outcomes to observed number of outcomes [E/O]) of the all-cause mortality submodel **eTable 4.** Lung cancer screening eligibility by removing race from LYFS-CT submodels, or by counterfactual-eligibility setting race/ethnicity to “White” in LYFS-CT submodels
eReferences

Supplement 2.
Data Sharing Statement
