# Supplementary material for: Methods for Using Race and Ethnicity in Prediction Models for Lung Cancer Screening Eligibility
Source: JAMA Netw Open. 2023 Sep 18;6(9):e2331155. doi: 10.1001/jamanetworkopen.2023.31155 (PMC10507484; doi:10.1001/jamanetworkopen.2023.31155)
Supplement: Supplement 1. — eMethods. eTable 1. Data sources used to develop and validate the lung cancer death risk and all-cause mortality models eTable 2. AUC of LYFS-CT submodels for risk of lung-cancer death and risk of all-cause mortality eTable 3. Calibration (ratio of expected number of outcomes to observed number of outcomes [E/O]) of the all-cause mortality submodel eTable 4. Lung cancer screening eligibility by removing race from LYFS-CT submodels, or by counterfactual-eligibility setting race/ethnicity to “White” in LYFS-CT submodels eReferences [file jamanetwopen-e2331155-s001.pdf]

Supplemental Online Content

Landy R, Gomez I, Caverly TJ, et al. Methods for using race and ethnicity in prediction models for lung cancer screening eligibility in the US population. *JAMA Netw Open*. 2023;6(8):e2331155. doi:10.1001/jamanetworkopen.2023.31155

eMethods
eTable 1. Data sources used to develop and validate the lung cancer death risk and all-cause mortality models
eTable 2. AUC of LYFS-CT submodels for risk of lung-cancer death and risk of all-cause mortality
eTable 3. Calibration (ratio of expected number of outcomes to observed number of outcomes [E/O]) of the all-cause mortality submodel
eTable 4. Lung cancer screening eligibility by removing race from LYFS-CT submodels, or by counterfactual-eligibility setting race/ethnicity to “White” in LYFS-CT submodels
eReferences

This supplemental material has been provided by the authors to give readers additional information about their work.

© 2023 Landy R et al. *JAMA Network Open.*

eMethods

Review of the Life-Years From Screening-CT (LYFS-CT) model (summarized from Cheung et al.[1])

Rationale for using individualized life-years gained rather than individualized risk for screening eligibility
Those at highest risk may not also be those with the greatest life-gained from screening. Risk-based lung-cancer screening preferentially includes older heavy-smokers with multiple comorbidities who have reduced life-expectancy and reduced life-gained from screening.[2] Microsimulation analyses showed that use of risk-calculators may not appreciably increase the life-gained by a population.[3] As a result, some medical societies have hesitated to endorse risk-based lung-cancer screening[4], and the USPSTF declined to recommend it in the draft 2020 guidelines. Some guidelines require 5 years of life-expectancy for high-risk individuals to enter screening.[5]

Individualized life-years gained from screening incorporates both individualized risk and individualized life-expectancy into a single model. Unlike individualized risk, individualized life-gained explicitly quantifies into a single metric several implicit considerations: disease-risk, life-expectancy, comorbidity/performance status, and the probability of benefits/harms from screening. Life-gained is perhaps the single most important summary consideration for patients, clinicians, cost-effectiveness, and guidelines committees. Life-gained focuses on the benefit of screening, i.e. increase in life-expectancy, while reducing potential harms, which tend to be associated with lower life-expectancy (advanced age and comorbid conditions).[6, 7]

In a comparison of choosing the top 8.3M for lung-cancer screening by risk or life-gained, life-gained strategies preferentially included younger, current smokers, with fewer comorbid conditions.[1] Those included for screening in life-gained but not risk-based selection had substantially higher gain in life-expectancy per prevented death versus to those included in risk-based selection alone. Those included in life-gained, but not risk-based, eligibility averaged age 59 with 0.75 average number of comorbidities. In contrast, those in risk-based, but not life-gained-based, eligibility averaged age 75 with 3.7 average number of comorbidities.

Calculating individualized life-years gained from screening: Life-Years From Screening-CT (LYFS-CT) model
LYFS-CT is composed of two submodels: one for individualized risk of lung-cancer death, and the other for risk of all-cause mortality. The submodel for risk of lung cancer death in the absence of CT screening is the previously validated Lung Cancer Death Risk Assessment Tool (LCDRAT).[8] LCDRAT has been validated in 5 cohorts, and is the only risk model for lung-cancer screening that has been validated using US nationally representative data, the NHIS.[8, 9] The submodel for risk of all-cause mortality was a Cox model that used the lung cancer risk factors in LCDRAT (except for family history of lung cancer), but also included 12 comorbidities highly predictive of mortality.[1] This model was trained on NHIS data and independently validated with different NHIS data.[1] See **Table 2** for LYFS-CT model parameters. We note that predictors of lung cancer death reflect predictors of lung cancer incidence and the stage at which lung cancer is diagnosed, as well as access to treatment. The hazard ratios for lung cancer incidence for each race/ethnicity are very similar to the hazard ratios for lung cancer death[10].

We assumed that, in the presence of NLST CT lung screening, there would be a 20.4% reduction in lung cancer mortality over 5 years, and no impact on other causes of mortality, as observed in the NLST. The life gained from screening is the difference in survival probabilities in the presence/absence of CT screening, integrated from the current age to a maximum attainable age. Deaths prevented in an NLST-like screening program accrue over a fixed time-period (5 years), while the gain in life-expectancy accrues both over the first 5 years and beyond due to a greater probability of survival to an older age.

The LYFS-CT model is available in our *lcmodels* R package and Excel spreadsheet[11].

© 2023 Landy R et al. *JAMA Network Open.*

Multiple imputation in NHIS 1997-2001

We used 5 multiple imputation datasets to account for missing information on race/ethnicity, education, BMI, quit years (years since quitting smoking), cigarettes smoked per day, and family history of lung cancer in NHIS 1997-2001, which was used to model competing risk of death in LCDRAT, the lung cancer death risk model. Details on the level of missing data for each variable for each year are provided in eTable 6 of Katki et al[10], with a summary provided here. The percentage of missing values were race/ethnicity – 1.2%; education – 0.4%; BMI – 2.3%; quit years: <0.1%; cigarettes smoked per day: 49.3% (only current smokers were asked, so smoking intensity was systematically missing for former smokers). Additionally, family history of lung cancer was only asked in 2000. Given its low prevalence (5.5%), we assumed that individuals with missing data for this variable had no family history of lung cancer.

For each variable with missing data, imputed values were obtained by randomly sampling from the known values among the subset of the NHIS validation cohort matched on sex, age (in 5-year age groups), and lung cancer death status. Applying this imputation approach for missing smoking intensity in former smokers could be problematic if cigarettes smoked per day when a regular smoker differ for former and current smokers. To investigate this issue, smoking intensity was assessed by smoking status in the 2005 NHIS, when data on cigarettes smoked per day was collected from both current and former smokers. After adjustment for age and sex, differences in smoking intensity between current and former smokers remained (see eTable 7 in the supplement to Katki et al[10]). The imputation procedure was therefore modified for cigarettes smoked per day in former smokers, setting the imputed value equal to the sum of the randomly sampled smoking intensity from the matched 1997-2001 NHIS cohort of current smokers and the age- and sex-specific mean difference between the 2005 NHIS former and current smokers. We note that we could not impute directly from the 2005 NHIS survey because this would prohibit matching on lung cancer death status.

Multiple imputation in NHIS 1997-2014

The same method for carrying out 5 imputations was used as in the section 'Multiple imputation in NHIS 1997-2001' above. The levels of missing data in NHIS 1997-2014 are as follows:

Covariate	Percent missing
BMI	2.9%
Education	0.6%
Race/ethnicity	0.0%
Age started smoking (used to calculate pack-years)	3.9%
Quit-years	1.1%
Cigarettes per day[1]	54.2%
Health problem requiring special equipment	0.0%
Liver condition in past year	0.1%
Emphysema	0.1%
Diabetes	0.1%
Weak/failing kidneys in the past year	0.1%
Prior cancer	0.1%
Prior stroke	0.1%
Prior myocardial infarction	0.2%
Coronary heart disease	0.3%
Heart disease	0.2%
Chronic bronchitis in past year	0.1%
Hypertension	0.2%
Angina pectoris	0.3%

[1] Individuals who formerly smoked were only asked the number of cigarettes smoked per day in 2000, 2005 and 2010

© 2023 Landy R et al. *JAMA Network Open.*

Multiple imputation in NHIS 2015-2018

The National Health Interview Survey (NHIS) is an annual survey of the civilian non-institutionalized population of the United States, which is carried out by the Centers for Disease Control and Prevention (CDC)[12]. We used data from 2015-2018, when a total of 118,859 individuals aged ≥18 years were interviewed. Each individual was assigned a weight, so the full survey weights up to the US population that year. We consider anyone who had smoked at least 100 cigarettes in their life to have ever smoked. Anyone who had never smoked, or who did not report whether they were a current, former, or never smoker was excluded from the analysis, as was anyone with a prior lung cancer, or whose baseline interview was completed by proxy. By these criteria, there were 25,601 adults aged 50-80 years who had ever smoked, representing 41.7 million individuals in the US who had ever smoked.

We used 5 multiple imputation datasets to account for missing data on BMI, race, education, quit years, and cigarettes smoked per day. The percentages of missing data among individuals aged 50-80 years who had ever smoked were: BMI: 2.9%, race: 1.9%, education: 0.4%, quit years: 0.4%. Current smokers had 1.7% missing data on cigarettes per day. Individuals who had formerly smoked were only asked about their cigarettes per day in 2015; therefore 76.1% of these individuals were missing this information. For each variable with missing values, an imputed value was randomly drawn from the observed values, conditional on sex and 10-year age category, and, for cigarettes smoked per day, on smoking status (former vs. current).

© 2023 Landy R et al. *JAMA Network Open.*

eTable 1: Data sources used to develop and validate the lung cancer death risk and all-cause mortality models.

Model	Data Source	Population	N (unweighted)	Follow-up
**Lung cancer death model**				
Model development	PLCO control arm	Individuals aged 55-74 who ever smoked	39,180	31 December 2009
Model validation	NHIS 1997-2001	Individuals aged 50-80 who ever smoked, without prior lung cancer, with known cause of death	28,232	31 December 2006
**All-cause mortality model**				
Model development	NHIS 1997-2014: odd years	Individuals aged 40-80 who ever smoked	74,842	31 December 2015
Model validation	NHIS 1997-2014: even years	Individuals aged 40-80 who ever smoked	72,199	31 December 2015
**Projections**	NHIS 2015-2018	Individuals aged 50-80 who ever smoked	25,601	n/a

© 2023 Landy R et al. *JAMA Network Open.*

eTable 2: AUC of LYFS-CT submodels for risk of lung-cancer death and risk of all-cause mortality. 95% confidence intervals are in parenthesis.

	N lung cancer deaths	AUC for standard LCDRAT (95% CI)	AUC for LCDRAT without race/ethnicity (95% CI)	N all-cause deaths	AUC for standard overall mortality model used in LYFS-CT (95% CI)	AUC for overall mortality model used in LYFS-CT, without race/ethnicity (95% CI)
**Total**	673	0.77 (0.75, 0.79)	0.77 (0.75, 0.79)	30462	0.86 (0.85, 0.86)	0.86 (0.85, 0.86)
**Self-reported race/ethnicity**						
African-American	98	0.79 (0.73, 0.85)	0.79 (0.73, 0.85)	3424	0.84 (0.83, 0.85)	0.84 (0.83, 0.85)
Asian-American	4	0.53 (0.06, 0.95)	0.53 (0.06, 0.95)	527	0.84 (0.79, 0.87)	0.84 (0.79, 0.87)
Hispanic-American	31	0.79 (0.67, 0.87)	0.79 (0.67, 0.87)	2658	0.82 (0.80, 0.84)	0.82 (0.80, 0.84)
White	534	0.76 (0.73, 0.79)	0.76 (0.73, 0.79)	18111	0.86 (0.86, 0.87)	0.86 (0.86, 0.87)

© 2023 Landy R et al. *JAMA Network Open.*

eTable 3: Calibration (ratio of expected number of outcomes to observed number of outcomes [E/O]) of the all-cause mortality submodel. 95% confidence intervals are in parentheses.

	E/O for current all-cause mortality model (95% CI)	E/O for all-cause mortality model without race/ethnicity (95% CI)
All-cause mortality risk - quintile 1 (lowest)		
Overall	0.98 (0.80, 1.20)	0.98 (0.80, 1.20)
African-American	1.14 (0.53, 2.46)	0.76 (0.41, 1.38)
Asian-American	0.83 (0.35, 1.99)	0.76 (0.32, 1.85)
Hispanic-American	0.50 (0.32, 0.78)	0.57 (0.34, 0.94)
White	1.12 (0.88, 1.43)	1.13 (0.88, 1.44)
All-cause mortality risk - quintile 2		
Overall	1.04 (0.94, 1.16)	1.06 (0.95, 1.19)
African-American	1.04 (0.75, 1.45)	0.92 (0.71, 1.18)
Asian-American	0.79 (0.47, 1.31)	1.36 (0.62, 2.98)
Hispanic-American	0.85 (0.65, 1.11)	1.03 (0.75, 1.40)
White	1.08 (0.95, 1.23)	1.08 (0.95, 1.23)
All-cause mortality risk - quintile 3		
Overall	1.05 (0.97, 1.13)	1.03 (0.96, 1.11)
African-American	1.07 (0.90, 1.27)	0.90 (0.76, 1.06)
Asian-American	0.85 (0.56, 1.27)	0.93 (0.64, 1.34)
Hispanic-American	0.95 (0.76, 1.18)	0.94 (0.77, 1.15)
White	1.07 (0.98, 1.16)	1.07 (0.98, 1.17)
All-cause mortality risk - quintile 4		
Overall	1.00 (0.95, 1.04)	1.00 (0.95, 1.04)
African-American	1.04 (0.91, 1.18)	0.85 (0.75, 0.95)
Asian-American	0.90 (0.69, 1.18)	1.35 (0.95, 1.91)
Hispanic-American	0.97 (0.83, 1.13)	1.14 (0.98, 1.34)
White	1.00 (0.95, 1.05)	1.00 (0.95, 1.05)
All-cause mortality risk - quintile 5 (highest)		
Overall	0.97 (0.95, 0.98)	0.97 (0.95, 0.98)
African-American	1.02 (0.98, 1.07)	0.93 (0.89, 0.97)
Asian-American	0.98 (0.85, 1.13)	1.14 (0.99, 1.31)
Hispanic-American	1.03 (0.97, 1.11)	1.14 (1.07, 1.22)
White	0.96 (0.94, 0.97)	0.96 (0.94, 0.98)

© 2023 Landy R et al. *JAMA Network Open.*

eTable 4: Lung cancer screening eligibility by removing race from LYFS-CT submodels, or by counterfactual-eligibility setting race/ethnicity to "White" in LYFS-CT submodels.

	Group	Number eligible	% eligible among all ever-smokers 50-80yrs	difference versus standard LYFS-CT model	% relative difference versus standard LYFS-CT model	Average life-days gained by LYFS-CT among the newly eligible	Average life-days gained by LYFS-CT among the newly ineligible
Standard LYFS-CT model	Total	7,574,448	17%				
	African-American	1,057,277	25%				
	Asian-American	93,247	7%				
	Hispanic-American	217,074	6%				
	White	6,206,850	18%				
LYFS-CT$_{NoRace}$	Total	7,608,880	17%	34,432	0.5%		
	African-American	643,463	15%	-413,813	-39.1%	n/a	18.51
	Asian-American	161,138	13%	67,891	72.8%	13.91	n/a
	Hispanic-American	450,678	12%	233,604	107.6%	13.53	n/a
	White	6,353,600	18%	146,750	2.4%	15.95	16.45
LYFS-CT$_{NoRaceACM}$	Total	7,632,524	17%	58,075	0.8%		
	African-American	1,193,543	28%	136,267	12.9%	15.54	n/a
	Asian-American	68,963	5%	-24,285	-26.0%	n/a	17.38
	Hispanic-American	186,277	5%	-30,797	-14.2%	n/a	16.70
	White	6,183,741	18%	-23,109	-0.4%	16.16	16.24
LYFS-CT$_{Counterfactual}$	Total	7,845,389	18%	270,941	3.6%		
	African-American	1,057,277	25%	0	0%	n/a	n/a
	Asian-American	160,057	13%	66,809	71.6%	13.94	n/a
	Hispanic-American	421,206	11%	204,132	94.0%	13.81	n/a
	White	6,206,850	18%	0	0%	n/a	n/a
LYFS-CT$_{CounterfactualACM}$	Total	7,712,922	17%	138,473	1.8%		
	African-American	1,195,750	28%	138,473	13.1%	15.53	n/a
	Asian-American	93,247	7%	0	0%	n/a	n/a
	Hispanic-American	217,074	6%	0	0%	n/a	n/a
	White	6,206,850	18%	0	0%	n/a	n/a

LYFS-CT$_{NoRace}$: Race/ethnicity removed from both submodels
LYFS-CT$_{NoRaceACM}$: Race/ethnicity removed from only all-cause mortality submodel
LYFS-CT$_{Counterfactual}$: Counterfactual eligibility: set race/ethnicity to 'white' in both submodels
LYFS-CT$_{CounterfactualACM}$: Counterfactual eligibility: set race/ethnicity to 'white' in only the all-cause mortality submodel

© 2023 Landy R et al. *JAMA Network Open.*

eReferences

1. Cheung LC, Berg CD, Castle PE, Katki HA, Chaturvedi AK. Life-Gained-Based Versus Risk-Based Selection of Smokers for Lung Cancer Screening. *Annals of internal medicine*. Nov 5 2019;171(9):623-632. doi:10.7326/M19-1263

2. Howard DH, Richards TB, Bach PB, Kegler MC, Berg CJ. Comorbidities, smoking status, and life expectancy among individuals eligible for lung cancer screening. *Cancer*. Dec 15 2015;121(24):4341-7. doi:10.1002/cncr.29677

3. Kumar V, Cohen JT, van Klaveren D, et al. Risk-Targeted Lung Cancer Screening: A Cost-Effectiveness Analysis. *Annals of internal medicine*. Feb 6 2018;168(3):161-169. doi:10.7326/M17-1401

4. Mazzone PJ, Silvestri GA, Patel S, et al. Screening for Lung Cancer: CHEST Guideline and Expert Panel Report. *Chest*. Apr 2018;153(4):954-985. doi:10.1016/j.chest.2018.01.016

5. Veterans Health Administration. Lung cancer screening saves lives. Veterans Health Administration. March 2018, Updated March 2018. Accessed April 3, 2019, https://www.va.gov/health/newsfeatures/2018/march/ldct-screening-enhances-cancer-care-for-veterans.asp

6. Black WC. Importance of Individualized Decision Making for Lung Cancer Screening. *Radiology*. Oct 2018;289(1):225-226. doi:10.1148/radiol.2018181220

7. Aberle DR, Adams AM, Berg CD, et al. Reduced lung-cancer mortality with low-dose computed tomographic screening. *N Engl J Med*. Aug 4 2011;365(5):395-409. doi:10.1056/NEJMoa1102873

8. Katki HA, Kovalchik SA, Berg CD, Cheung LC, Chaturvedi AK. Development and Validation of Risk Models to Select Ever-Smokers for CT Lung Cancer Screening. *JAMA*. Jun 07 2016;315(21):2300-11. doi:10.1001/jama.2016.6255

9. Katki HA, Kovalchik SA, Petito LC, et al. Implications of Nine Risk Prediction Models for Selecting Ever-Smokers for Computed Tomography Lung Cancer Screening. *Annals of internal medicine*. Jul 3 2018;169(1):10-19. doi:10.7326/M17-2701

10. Katki HA, Kovalchik SA, Berg CD, Cheung LC, Chaturvedi AK. Development and validation of risk models to select ever-smokers for CT lung cancer screening. *JAMA*. 2016;315(21):2300-2311.

11. Cheung LC, Katki HA. lcmodels: R package for predictions from published lung cancer models. Accessed April 5, 2019, https://dceg.cancer.gov/tools/risk-assessment/lcmodels

12. CDC/National Center for Health Statistics. National Health Interview Survey. 12 January 2020, https://www.cdc.gov/nchs/nhis/

© 2023 Landy R et al. *JAMA Network Open.*
