# Supplementary material for: Methods for Using Race and Ethnicity in Prediction Models for Lung Cancer Screening Eligibility
Source: JAMA Netw Open. 2023 Sep 18;6(9):e2331155. doi: 10.1001/jamanetworkopen.2023.31155 (PMC10507484; doi:10.1001/jamanetworkopen.2023.31155)
Supplement: Supplement 2. — Data Sharing Statement [file jamanetwopen-e2331155-s002.pdf]

Data Sharing Statement

Landy. Methods for Using Race and Ethnicity in Prediction Models for Lung Cancer Screening Eligibility. *JAMA Netw Open*. Published September 18, 2023.
doi:10.1001/jamanetworkopen.2023.31155

Data
Data available: Yes
Data types: Deidentified participant data
How to access data: https://cdas.cancer.gov/
When available: beginning date: 04-10-2023

Supporting Documents
Document types: None

Additional Information
Who can access the data: researchers whose proposed use of the data has been approved
Types of analyses: any approved research
Mechanisms of data availability: after approval of a proposal
